# Unfolded protein response (UPR) integrated signaling networks determine cell fate during hypoxia

**DOI:** 10.1186/s11658-020-00212-1

**Published:** 2020-03-13

**Authors:** Sylwia Bartoszewska, James F. Collawn

**Affiliations:** 1 Department of Inorganic Chemistry, Medical University of Gdansk, Gdansk, Poland; 2 Department of Cell, Developmental and Integrative Biology, University of Alabama at Birmingham, Birmingham, USA

**Keywords:** ER-stress, Angiogenesis, Hypoxia-reoxygenation injury, Ischemia, Cell fate determination, UPRmt

## Abstract

During hypoxic conditions, cells undergo critical adaptive responses that include the up-regulation of hypoxia-inducible proteins (HIFs) and the induction of the unfolded protein response (UPR). While their induced signaling pathways have many distinct targets, there are some important connections as well. Despite the extensive studies on both of these signaling pathways, the exact mechanisms involved that determine survival versus apoptosis remain largely unexplained and therefore beyond therapeutic control. Here we discuss the complex relationship between the HIF and UPR signaling pathways and the importance of understanding how these pathways differ between normal and cancer cell models.

**This article was specially invited by the editors and represents work by leading researchers.**

## Introduction

Aerobic organisms employ critical control strategies to ensure proper oxygen supply through various physiological and metabolic cellular signaling networks. The inability to meet cellular oxygen demands, termed hypoxia, results in the activation of specific cellular stress responses [1, 2]. Hypoxic stress induces global gene expression changes in order to help cells adapt and survive by altering the cell’s metabolic and angiogenic pathways and restoring oxygen homeostasis [3–10]. If these repair and adaptive mechanisms fail, cells modify their gene expression profiles to induce programmed cell death [11–16]. Although active hypoxia signaling networks are necessary during embryogenesis and development [17–19], hypoxic conditions either diminish normally, or they contribute to pathological events in mature organisms [20–23].

Efficient activation of hypoxia signaling and angiogenesis is critical, for example, after stroke [24], myocardial infarction [25], and other ischemic events [26–29]. Alternatively, metabolic adaptation to low oxygen levels and the related tissue revascularization allows for the survival and progression of the majority of human tumors [30–32], and contributes to macular degeneration [33–36], glaucoma progression [37], and diabetic retinopathy [38–41]. Thus, the discovery and development of therapeutic strategies exploiting hypoxia-related cellular networks are of great interest to modern medicine, as evidenced by the awarding of the 2019 Nobel Prize in Physiology or Medicine to Drs. Semenza, Ratcliffe, Kaelin on their research into how cells detect oxygen and react to hypoxia [42–46].

The main goal of the cellular response to hypoxia is to promote cell survival and restore oxygen homeostasis. This goal, however, is accompanied by deregulation of cellular organelle changes in mitochondria and endoplasmic reticulum (ER) function that are reflected in perturbations in protein folding and trafficking [4, 47–53]. Erratic protein folding activates another specific stress response pathway, the unfolded protein response (UPR). The UPR promotes cellular survival by restoring endoplasmic and mitochondrial homeostasis through its distinct signaling networks [54–56], but if unsuccessful, the UPR induces cell death [57–59].

Although activation of the UPR supports surviving hypoxia, it can also impair cellular survival [60]. The ER, for example, is responsible for folding and maturation of transmembrane and secretory proteins [61–69] that include proangiogenic receptors and ligands such as vascular endothelial growth factor (VEGF) and erythropoietin (EPO) that are critical for hypoxia-induced angiogenesis and erythropoiesis, respectively [70–72]. Thus, although underappreciated, understanding mutual crosstalk between these stress response pathways is important for understanding and developing therapeutic interventions in cardiovascular diseases and cancer. Nevertheless, despite the extensive studies on both of these stress responses, the resulting consequences of their collective activation remain largely unexplained and are mainly limited to in vitro cell culture-based models. In this review, we summarize these two cell survival pathways and the implications of UPR involvement in the hypoxia cellular response pathway.

## Hypoxia-inducible factor responses to hypoxia

**Fig. 1:**
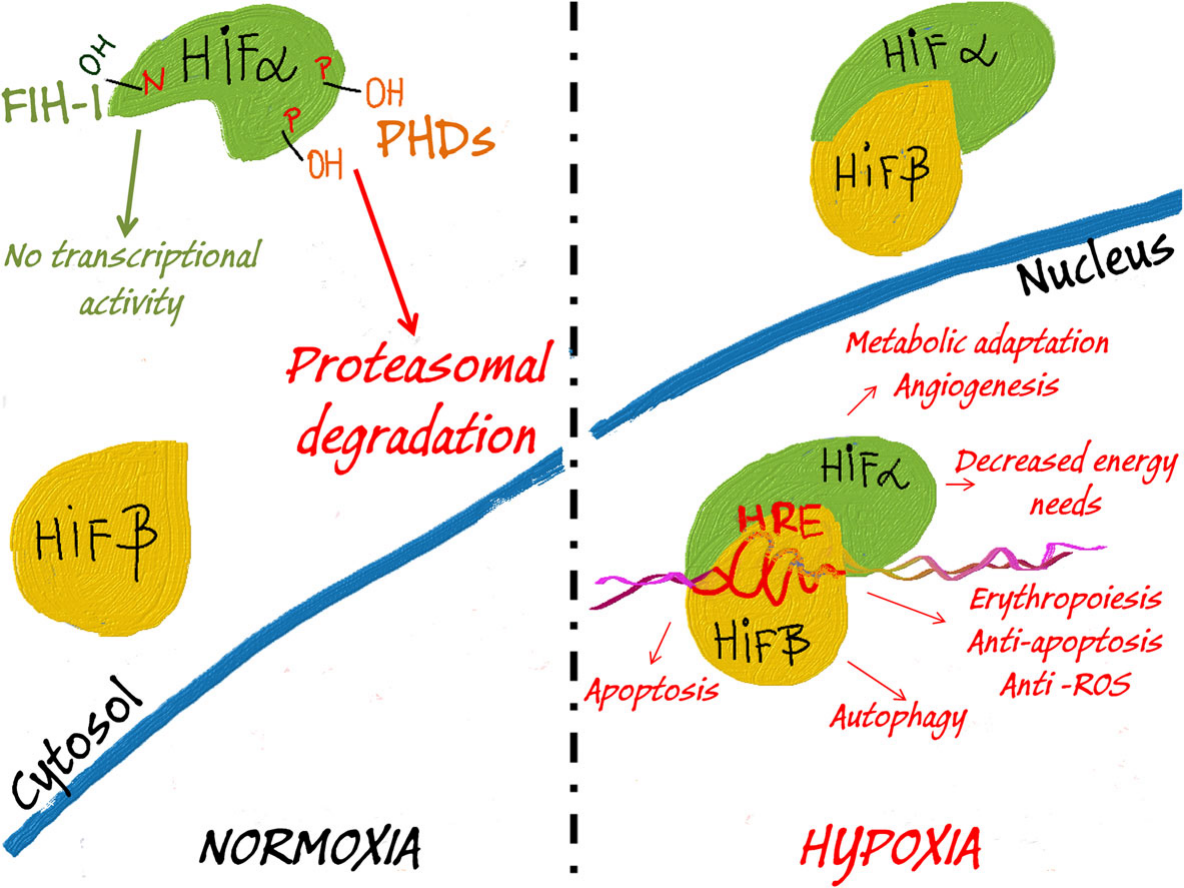
Oxygen availability regulates HIF signaling. In normoxia, proline (P) residues on HIFα subunits are hydroxylated by PHDs that marks them for proteasomal degradation. Additionally, FIH-1 mediates hydroxylation of asparagine residues (N) on HIFα to prevent HIF transcriptional activity. Hypoxia impairs the ability of PHDs and FIH-1 to hydroxylate the HIFα subunits, and thus results in the accumulation of this subunit and its heterodimerization with the stable HIFβ subunits. In the nucleus, the HIFα/β complex binds to HRE elements in the HIF target genes and governs their expression in order to adapt the cells to hypoxic conditions

The unmet cellular oxygen demand is reflected by the accumulation of functional heterodimeric α/β-subunit complexes of specific transcription factors called hypoxia-inducible factors (HIFs) [42–46]. HIFs mediate both the adaptive and apoptotic responses to hypoxia through transcriptional modulation of genes containing their specific target sequences that are termed hypoxia-response elements (HREs) [7, 73–77]. If cells are sufficiently supplied with the oxygen, the formation of active HIF complexes is inhibited by the limited availability of the alpha (α) subunits. Under normal oxygen pressure (normoxia), HIF-α subunits undergo oxygen-dependent post-translational modifications by proline-hydroxylases (PHDs) that mark these subunits for subsequent proteasomal degradation [42–46]. Furthermore, during normoxia there is another oxygen-dependent post-translational modification of α-subunits that is mediated by the factor inhibiting HIF (FIH) which impairs HIF transcriptional activity (Fig. 1) [78]. In contrast, the cellular levels of HIF-β subunits are oxygen independent [42–46]. During hypoxia, the post-translational modifications of HIF-α subunits are inhibited and lead to accumulation of the alpha subunit and the transcriptionally active HIF-αβ hetero-complexes. Despite the fact that HIF-1α is considered a major mediator of HIF signaling in higher metazoans, other tissue specific isoforms of α-subunits, HIF-2α and HIF-3α, are also known to participate in the cellular responses to hypoxia [7, 79–84].

## The pro-survival pathway

During hypoxia, HIFs execute pro-survival transcriptomic strategies that allow cells to sustain energy levels via utilization of less efficient non-oxidative energy production. To sustain energy levels, HIFs upregulate glucose transporter genes and glycolytic enzymes, and inhibit oxidative phosphorylation (1) by preventing the conversion of pyruvate to acetyl-Co-A, (2) by reducing glucose oxidation and (3) by inhibiting β-oxidation of fatty acids [85–88]. Importantly, hypoxia-related utilization of this alternate metabolic pathway is accompanied by a HIF-mediated activation of the mechanisms that allow for a more efficient utilization of the anaerobic glycolytic pathway and that minimize its negative impact on the cell. The goal is to increase the electron transfer efficiency and to reduce reactive oxygen species (ROS) production. HIF-1 also regulates cytochrome c oxidase (COX) subunit composition to optimize the efficiency of respiration during hypoxia and to reduce ROS by promoting ROS scavenging pathways [89, 90]. Furthermore, since anaerobic glycolysis results in increased proton release, HIF-1 induces the expression of carbonic anhydrase 9 (*CA*-*IX*) and monocarboxylate transporter 4 (*MCT4*) to counteract acidosis by regulating intracellular pH [91, 92].

Since non-oxidative energy production of the cellular levels of ATP is less efficient than oxidative phosphorylation, HIFs activate pathways to decrease the cell’s energy needs. To accomplish this, HIFs selectively suppress translation and therefore decrease total protein production [93–95] and induce induction of autophagy [96, 97] and mitophagy [95, 98]. Notably, the mTOR pathway also reduces protein synthesis and cell growth and induces autophagy via a HIF-independent mechanism [48, 99].

In order to restore oxygen homeostasis and maintain the well-being of the endothelium, HIFs stimulate the expression of a number of angiogenic genes that include the vascular endothelial growth factor (*VEGF*) [9, 100], heme oxygenase-1 (*HMOX1*) [101], matrix metalloproteinases (*MMP*) *2* and *13* [102], the stem cell factor *OCT-3/4* [103, 104], angiopoietin 2 (*ANGPT2*) [105], stromal derived factor 1 (*SDF1*) [106], platelet-derived growth factor B (*PDGFB*) [107], placental growth factor (*PGF*) [108], and stem cell factor (*SCF* )[109] and endothelial nitric oxide synthase (*NOS3*) [110, 111]. While HIF-induced angiogenesis ensures increased blood flow to hypoxic tissues, the oxygen caring capacity of the blood is enhanced via HIF-dependent upregulation of erythropoietin [112–114]. Importantly, to secure proper cellular iron levels that are required for the efficient erythropoiesis, HIFs adjust the expression of transferrin as well as of other genes mediating iron homeostasis [115, 116]. Furthermore, EPO supports anti-apoptotic proteins and inhibits caspase activity [117–119].

## The UPR pathway responses to hypoxia

The fundamental function of the cellular response to hypoxia is surviving precarious conditions and restoring oxygen homeostasis. Hence, despite the HIF-related mechanisms to reduce the negative effects of anaerobic glycolysis and the reduced energy availability, this metabolic switch eventually disturbs cellular homeostasis. This energy deficiency limits the activity of ATP-dependent processes such as maintenance of ion homeostasis and the related redox potential, and limits protein and lipid synthesis, and post-translational protein folding capabilities due to the impaired disulfide-bond formation and ROS activity [4, 120–124]. All of these factors can disturb endoplasmic reticulum homeostasis (termed as ER stress), and lead to the accumulation of unfolded or misfolded proteins in the ER [125]. The accumulation of misfolded proteins activate another specialized stress response signaling pathway called unfolded protein response (UPR) [125]. During hypoxia, there are critical changes in mitochondrial function that lead to elevated ROS levels. Furthermore, the proper folding of mitochondria-encoded as well as the import and corresponding refolding of mitochondrial nucleus-encoded proteins are crucial for mitochondrial function [126, 127]. Hence, prolonged hypoxia will eventually result in perturbations in mitochondrial protein folding and activation of a related specific stress response mechanism termed the mitochondrial unfolded protein response (UPRmt) [126, 128–130].

## The three UPR signaling pathways

**Fig. 2:**
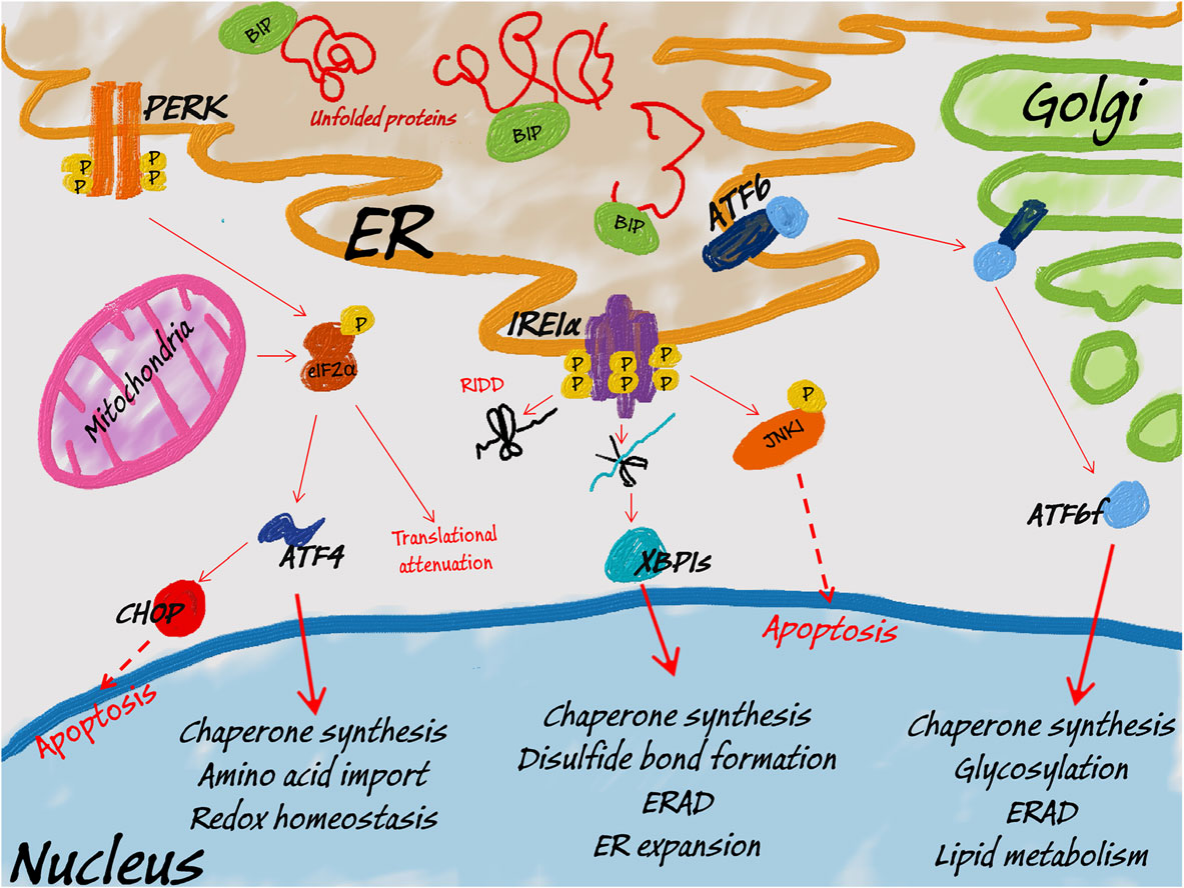
UPR and UPRmt signaling. Upon buildup of misfolded/unfolded proteins in ER, BIP is released from ER membrane to induce PERK dimerization and its subsequent autophosphorylation. Activated PERK phosphorylates the eIF2α, leading to global translation attenuation. Some transcripts, however, including ATF4 remain preferably translated. ATF4 provides the transcriptional signal to restore ER homeostasis, however, it can also induce proapoptotic CHOP. Similarly, accumulation of unfolded proteins in mitochondria leads to PERK activation and the induction of ATF4 signaling (UPRmt). Upon its dissociation from BIP, IRE1α undergoes oligomerization and autophosphorylation and thus gains endoribonuclease activity. To decrease the ER load, activated IRE1α degrades mRNAs and miRNAs (RIDD). IRE1α also performs splicing of XBP1 mRNA to release transcriptionally active XBP1s. XBP1s activates a transcriptional program to restore ER homeostasis. Alternatively, IRE1α can activate a proapoptotic kinase JNK1. Finally, BIP dissociation allows ATF6 translocation to Golgi, where cleavage of this protein results in release of transcriptionally active ATF6f. ATF6f activates a transcriptional program to restore ER homeostasis and support ERAD

Controlling ER homeostasis relies on the interplay between three signaling pathways of UPR that are initiated by three distinctive transmembrane sensors [125]. Buildup of unfolded/misfolded proteins in the ER induces a higher demand for chaperone proteins that include glucose-regulated protein 78 (GRP78 also known as BiP (binding immunoglobin protein) [57]. BiP initiates the UPR by dissociating from luminal domains of three proteins, protein kinase RNA-like endoplasmic reticulum kinase (PERK), the inositol-requiring enzyme 1α (IRE1α), and with activating transcription factor 6 (ATF6) [57]. Upon BiP release, PERK and IRE1α are activated via multimerization and trans-autophosphorylation, whereas ATF6 is translocated to the Golgi apparatus where it is proteolytically processed to a cytoplasmically soluble and active ATF6f (p50) transcription factor (Fig. 2) [131–133]. This activation cascade results in three distinctive UPR signaling pathways/axes that are mediated by the PERK, IRE1α and ATF6 sensors.

Active PERK phosphorylates the alpha subunit of the eukaryotic initiation factor eIF2, and this initiates the selective translation of certain proteins and repressing the translation others during stress conditions. Some of the selected proteins include activating transcription factor 4 (ATF4), growth arrest and DNA damage inducible protein (GADD34), and CCAAT/enhancer binding homologous protein (CHOP) [57, 134, 135]. ATF4 modulates the expression of genes involved in amino acid biosynthesis, anti-oxidative responses, protein folding and in maintaining redox homeostasis [136]. Importantly, GADD34 mediates the dephosphorylation of eIF2, thus allowing the restoration of the protein synthesis upon stress recovery [137]. If the stress is persistent, ATF4 can also facilitate autophagy and stimulate transcription of the proapoptotic CHOP to induce cell death (Fig. 2) [138, 139].

Active IRE1α reduces protein synthesis through the degradation of selected mRNAs in a process referred to as regulated IRE1-dependent decay (RIDD) [140]. Notably, IRE1α endoribonuclease activity generates the active spliced isoform of the X-box binding-protein transcription factor (XBP1s) [141]. XBP1s modulates gene expression by increasing the ER’s folding capacity. XBP1s also promotes the expression of proteins that are involved in ER membrane biosynthesis, disulfide bond formation, as well as increasing the expression of chaperones and proteins involved in ER-associated degradation (EDEM) and vesicular trafficking [141–143]. Furthermore, IRE1α kinase activity activates Janus N-terminal kinase (JNK) in order to activate the inflammatory response and to promote autophagy and apoptosis [140, 144] (Fig. 2).

ATF6f, on the other hand, initiates a transcriptional program to restore ER homeostasis and that includes the induction of BIP expression, promoting protein chaperone and lipid synthesis, stimulating ER-degradation, and enhancing N-glycosylation [145, 146]. ATF6f also induces *CHOP* expression and thus contributes to UPR-related cell death [147, 148] (Fig. 2). Notably however, a recent report has shown that IRE1α activation can deactivate the ATF6f pathway [149].

Despite the fact that the UPR usually mediates cell death by activating the intrinsic apoptotic pathway, recent reports indicate that during unresolved ER stress, there is strong activation of the UPR that can lead to activation of programmed-necrosis pathways such as necroptosis [149–154]. Activation of these cell death pathways usually involves PERK signaling and is associated with a rapid depletion of intracellular ATP and a rapid release of ER-stored calcium [149–154]. Notably, the necroptosis pathway has been involved in modulation of both HIF-signaling and key glycolytic enzymes that include pyruvate dehydrogenase. This results in the enhancement of aerobic respiration and ROS generation, and thus can lead to impaired cellular adaptation to hypoxia [155–158]. That being said, the origins and role of necroptosis in both the UPR and the hypoxia response will require further studies.

## Mitochondrial stress responses

Since mitochondria are separated from the cytosol and ER by their outer and inner membranes, they have to rely on their own stress response mechanisms for translating and folding proteins encoded in their genomes as well as refolding the imported nuclear-encoded proteins [126, 127]. In order to maintain their protein homeostasis, these organelles have a specific set of chaperones that includes heat shock protein 60 (HSP60) and LON peptidase 1 [159–161]. Notably, it has been reported that events that lead to accumulation of unfolded/misfolded proteins in the mitochondria, or in impairment of energy dependent mitochondrial protein import, or in disturbances in mitochondrial protein synthesis and folding lead to the activation of a mitochondrial UPR (UPRmt) [126, 128–130].

To recover and preserve mitochondrial function, UPRmt modulates the expression of both mitochondria and nuclear encoded genes [126, 128–130]. However, if the stress is persistent, the UPRmt can contribute to the activation of intrinsic apoptosis pathways [126, 128–130]. In *C. elegans*, properly functional mitochondria import and subsequently degrade the stress sensor protein called activating transcription factor associated with stress (ATFS-1) [162]. Upon stress, however, ATFS-1 import to mitochondria is impaired, and this transcription factor accumulates in nucleus and activates a transcriptional program to restore mitochondrial homeostasis through upregulation of mitochondrial chaperons and proteases as well as components of both the protein import machinery and ROS scavenger pathway [162].

Although the regulation of the mammalian UPRmt is poorly understood, it has been suggested that the import efficiency of activating transcription factors 5 and 4 (ATF5 and ATF4) can be sensors of mitochondrial protein disturbances [163, 164]. Upon stress, these transcription factors were shown to induce expression of mitochondrial chaperones and proteases. Furthermore, it has been shown that disturbances of mitochondrial protein homeostasis lead to activation of the PERK axis of the UPR, and this reduces global protein synthesis and selectively promotes expression of ATF4, ATF5 and the proapoptotic protein CHOP (Fig. 2) [126, 128–130, 165]*.* However, the molecular mechanisms underpinning the integrated feedback between the UPR and the UPRmt will require further study.

## The crosstalk between hypoxia and UPR in cancer versus normal cell models

Despite the fact that normal endothelial cells are the main effectors of the adaptive cellular response to hypoxia, the vast majority of current research regarding this signaling pathway is from cancer cells [31, 48, 166, 167]. The mainstream reports of the interplay between hypoxia and UPR are limited to cancer models as well [71, 72, 167–171]. Importantly, cancer progression and cancer cell survival often result from the deregulation of the cell fate decision mechanisms during both hypoxia and the UPR. Although hypoxia was shown to induce all three UPR signaling axes, and given their activation could also result from cancer cell-specific adaptations, it is important that the prosurvival consequences of the UPR need to be directly compared to normal cell types.

Hypoxia-related induction of BIP expression has been reported in both cancer and endothelial cells models [50, 110, 172–176]. This suggests that hypoxia-induced perturbations in ER may increase BIP demand in both cell types and promote UPR induction. Indeed, activation of PERK signaling is also observed in both cancer and normal cells including endothelial cells, regardless of the hypoxia model applied [170, 177–182]. PERK-mediated eIF2 phosphorylation was observed in cells within minutes after exposure to acute hypoxia (below 0.1% O_2_), whereas this reaction rate continuously declined with increasing oxygen concentrations [177]. Furthermore, activation of the PERK axis was also reported in transient (cyclic hypoxia) models that better resemble the fluctuating oxygen availability conditions that occur in solid tumors [183–187]. Hence, it can be concluded that the hypoxia-required reduction of energy demand is partially achieved via UPR-mediated translational attenuation. Notably, this pathway was shown to be deactivated during prolonged hypoxia (16 h) as shown by dephosphorylation of eIF2 that is probably due to a negative feedback loop with GADD34 [177, 188, 189]. During prolonged hypoxia, HIF-1 signaling is only partially sustained by the HIF-2 activity during the transition from HIF-1 to HIF-2 expression [7, 76, 77]. This would suggest that the activation of PERK axis can only be modulated by the HIF-1, whereas during prolonged hypoxia, HIF-2 mediates the translational repression via an alternate mechanism [167]. However, this hypothesis will require further study. Interestingly, the PERK pathway was also shown to inhibit HIF-1α translation and thus prevent HIF-1 signaling in cancer cells [190].

Besides attenuation of protein synthesis, the PERK pathway mediated by ATF4 activates genes supporting ER and mitochondrial homeostasis [126, 128–130, 165]. Notably, however, the PERK pathway can induce cell death through CHOP accumulation [191]. Although CHOP accumulation and the potential induction of apoptotic response were observed in some hypoxia experiments (including lung endothelial cells) [192–194], this protein and mRNA levels were much lower than those observed during ER stress [177]. Inhibition of the entire PERK axis during hypoxia, however, has more drastic effects on cell survival [177]. Furthermore, hypoxic PERK activation was shown to regulate carbonic anhydrase 9 (CA9) levels and thus is important for maintaining cellular pH [195, 196]. Importantly, CHOP also directly reduces the expression of the proangiogenic endothelial nitric synthase (*NOS3, eNOS)* [197]. The reduction of eNOS activity during hypoxia, however, may be required to prevent the uncoupling of this enzyme and the related ROS accumulation [198, 199]. Therefore, the evaluation of CHOP’s role in hypoxic cell survival requires careful consideration and further study [200].

The activation of IRE1 axis and the role of XBP1s during hypoxia remain less clear. Despite some functionally relevant accumulation of XBP1s that supported cancer cell survival and tumor growth studies in cancers cell lines exposed to hypoxia, this effect was observed in acute and moderate hypoxia only [177, 201–209]. In contrast, impairment of *XBP1* splicing under acute hypoxia was also reported [210]. Furthermore, although some potentially IRE1-related activity was observed in human pulmonary artery smooth muscle cells (PAMSCs), this did not result in direct hypoxia-induced XBP1s protein accumulation [211]. Hence, IRE1α involvement in cellular response may be very oxygen pressure- and cell type-specific and will require further studies in a wide range of primary endothelial cells [212–216]. Finally, although numerous known ATF6 transcriptional targets were shown to be elevated in some experimental models by prolonged hypoxia and ischemia [192, 217, 218], the general direct hypoxic activation of the ATF6 UPR axis has been convincingly presented [177]. Hopefully, the novel ATF6 pathway inhibitors, Ceapins [219], will be helpful in clarifying the role of this UPR branch during hypoxia.

The main function of adaptive HIF activity is with the induction of angiogenesis and erythropoiesis. The successful implementation of these cell rescue programs requires increased synthesis of proangiogenic factors (ligands and receptors) as well as increased erythropoietin production. All of these proteins fall into either the transmembrane or secretory proteins category, and as such they have to mature in the ER [52, 121, 220–223]. Hence, recovery from hypoxia absolutely depends on proper ER function [224]. Importantly, the PERK/ATF4 axis has been reported as a limiting factor for EPO production, and thus hypoxic UPR activation may limit adaptation to hypoxia [70].

In 2014, Karali and coworkers described the mechanism potentially linking HIF transcriptional activity with the activation of PERK, ATF6 and IRE1 pathways in human endothelial cells [225]. They reported prosurvival UPR activation in VEGF (a HIF transcriptional target) treated human umbilical vein endothelial cells (HUVECs) [226]. In these studies, the authors proposed a mechanism in which VEGF-dependent phosphorylation of vascular endothelial growth factor receptors (VGFRs) leads to phospholipase C (PLC) activation and release of ER calcium, which activates all three axes of the UPR [225]. The active UPR promotes transcriptional expression of numerous proangiogenic genes that include *VEGF* that can be induced directly by ATF6f, XBP1s and ATF4 [224, 227–237], and interleukin 8 that is induced by ATF4 [238, 239]. Furthermore, it has been suggested that this survival mechanism is mediated by XBP-1 through a direct interaction with HIF-1α protein [201]. Nevertheless, the extend of VEGF-related activation of UPR is rather poorly reflected in endothelial hypoxia models, and will require further study. In endothelial cells, it has been also reported that hypoxia-induced expression of very low density lipoprotein receptor (VLDLr) can contribute to the activation of the UPR cell death response [193, 194].

## The UPR and UPRmt

**Fig. 3:**
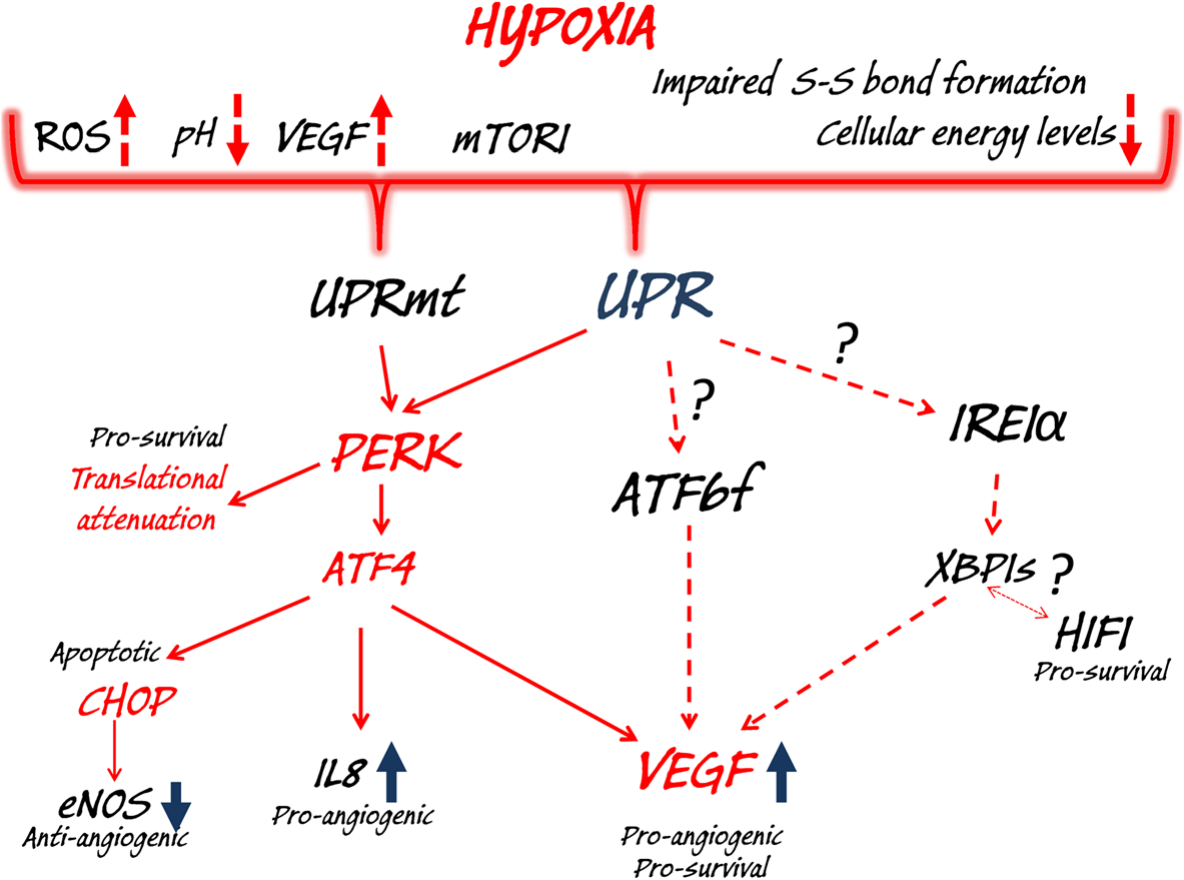
Hypoxia signaling and the related changes in cellular functions activate the UPR and UPRmt. During hypoxia, accumulation of misfolded/unfolded proteins in ER and mitochondria activate PERK signaling, and this contributes to both pro-survival (global translational arrest and induction of pro-angiogenic genes IL8 and VEGF) and apoptotic responses (induction of CHOP and inhibition of pro-angiogenic eNOS expression). Furthermore, in some models, hypoxia-related activation of ATF6 and IRE1α contributes to pro-survival and pro-angiogenic signaling. There also appears to be cooperation between XBP1s and HIF1 in pro-survival signaling

Collectively, despite a variety of ER stress-related mechanisms reported in cancer cells, exposure to hypoxia results mainly in the activation of the PERK axis that can also be co-regulated by UPRmt (Fig. 3). Notably, however, the dramatic activation of all arms of UPR was reported in anoxia and that by definition relates to the loss of control over the cellular metabolism and energy production due to a dramatic oxygen deficit [240]. Given that limitation, such as reports are hard to interpret since they were obtained from mixed cells populations that were undergoing the anoxic necrosis and had totally lost the ability to maintain their redox homeostasis.

By comparison, the limited activation of the UPR and UPRmt during moderate and prolonged hypoxia suggests that the HIFs successfully prevent extensive ROS formation and alleviate stress conditions. This statement is supported by the studies that have shown that HIF-1α knockouts cells subjected to hypoxia produce lethal levels of ROS [241] as well as by numerous reports demonstrating a negative correlation between cellular ROS levels and HIF-1 stabilization [87, 90, 242–244]. Remarkably, however, cumulating evidence demonstrates that rapid re-establishment of normal oxygen levels in hypoxic cells often results in extensive ROS production and leads to cellular damage that is referred to as hypoxia-reoxygenation injury and ischemia-reperfusion injury. Although ROS accumulate in hypoxic cells, their levels are dramatically compounded by this rapid re-introduction of oxygen [90, 241, 245, 246].

Importantly, both ischemia-reperfusion injury [247–259] and hypoxia-reoxygenation injury [260–271] are also known to be accompanied by ER stress that are related to hypoxia/reoxygenation-triggered depletion of ER calcium and ROS accumulation. Indeed, an extensive UPR and UPRmt activation occurred upon rapid restoring oxygen levels in human endothelial cells, cardiomyocytes and neurons [247–271]. Although the IRE1α and ATF6 axes were activated in some models similar to their activation to hypoxia [272, 273], the PERK pathway was the common and main axis of UPR activation after hypoxia/reoxygenation. Importantly, the UPR pathway was shown to be crucial for determining cell fate during hypoxia/reoxygenation, and therefore this pathway should be considered as a potential therapeutic target for ischemic and cardiovascular diseases.

Importantly, intermittent (cyclic hypoxia) results from chronic exposure of cells to cycles of hypoxia/reoxygenation and is a basic feature of sleep apnea. Cyclic hypoxia also clearly defines the development of the majority of solid tumors, which were also shown to be accompanied by chronic ER stress [274, 275]. Nevertheless, the majority of the hypoxia-induced UPR cancer research has been performed in continuous hypoxia models that display diminished UPR activation.

## Concluding remarks

The cellular response to hypoxia as well as the UPR are critical components of human pathologies and have become obvious therapeutic targets. Despite the continuous research to elucidate these complex molecular signaling pathways, however, the exact mechanisms that cells use to determine cell fate during stress remain mainly largely unexplained and beyond therapeutic control. Furthermore the current understanding of the molecular mechanisms underpinning the mutual interplay between cellular response to hypoxia and UPR remain very limited. Although hypoxia-related ER stress is well defined, the extent of the related UPR activation and its effects on angiogenesis and particularly cell survival are complicated and often contradictory. Several research cell model-related barriers need to be tested in order to gain better insight into physiological relevance of the hypoxic UPR before any type of intervention could be properly tested in vivo.

To date, the two dimensional in vitro cultures of cancer cells that are exposed to continuous hypoxia constitute the main research cell models. As mentioned, the cancer cell lines have developed specific genetic and epigenetic adaptations that allow them to adapt and survive both persistent ER-stress and hypoxia. Therefore, parallel research in matched normal, primary cells is needed to distinguish between the cancer-specific and cell line-specific signaling pathway mechanisms employed for cell fate decisions.

In solid tumors, for example, cancer cells usually benefit from endothelial angiogenesis, and thus despite sending angiogenic signals to endothelial cells, they alone not the right model for determining the extent of how hypoxia or the UPR impacts angiogenesis. Hence, the role of tumors’ endothelial cells and the development of representative models including both cancer and endothelial cells, is extremely important. Notably, the current development of high-throughput single-cell transcriptomics on organoids and 3D culture systems [276, 277] should contribute to the utilization of such a research approach.

Furthermore, solid tumors are persistently exposed to fluctuating oxygen levels (cyclic hypoxia) rather than chronic hypoxia [278–283]. Given the potent activation of the UPR during cycles of hypoxia/reoxygenation, it is plausible that majority of the cancer studies underestimate the UPR component of the response to hypoxia in these tumors. Moreover, human cells and tissues differ in their oxygen demands, and the fate of individual cells and the magnitude of the individual cellular stress responses are affected by the local levels of oxygen. This would suggest that the consequences of reoxygenation to the lower actual tissue oxygen levels (termed physoxia) should be considered as well [2].

Normal endothelial cells provide an alternative model to study hypoxia and the related UPR. The comparative analysis of endothelial cells from different vascular beds, for example, could provide novel insights into the physiological relevance of hypoxia-induced UPR. Many studies have been based on models where cells were exposed to persistent moderate continuous hypoxia (e.g., 12 or 24 h at 1% oxygen) that is physiologically irrelevant and therefore difficult to interpret.

Recent studies have identified novel post-transcriptional levels of regulation of cellular signaling pathways that depend upon the RNA interference pathway, and include mRNA modulation by microRNAs. Although, these short noncoding RNAs has been reported to be important modulators of both hypoxia and UPR [59, 77, 83, 143, 187, 284–294], their role in the interplay between these two signaling pathways remains limited. Of note in this regard, a recent report has shown that XBP1s induces antiangiogenic miR-153 during hypoxia in breast cancer cells [202, 203].

Further development of new experimental models, wide transcriptomic and proteomic approaches, as well as employment of novel specific inhibitors of UPR axes will eventually address some of these issues. Furthermore, numerous novel compounds and therapeutic strategies focusing on cancer and cardiovascular diseases are progressing through clinical trials that target either the hypoxic response or the UPR (https://clinicaltrials.gov/ct2/ and https://clinicaltrials.gov/ct2/, respectively) [294–298]. Nevertheless, the crosstalk between these stress pathways is rarely utilized in these putative therapies. For example, some of the anticancer agents are proteasome inhibitors (bortezomib and nelfinavir) that utilize the UPR pathways to decrease VEGF levels and thus directly inhibit tumor vasculature [299–302], whereas the ER chaperones inhibitor 17-AAG (geldanamycin) reduces the degree of adaptive HIF-1 signaling and thus stimulates hypoxic cell death [303, 304]. Finally, although the interplay between the hypoxia and the related UPR is just beginning to be appreciated, we are still very far from understanding their interrelated functions and therefore further research in this field will be critical for the development of future therapies.

## Data Availability

Not applicable.

## Abbreviations

ATF4: Activating transcription factor 4
ATF6: Activating transcription factor 6
BiP: Also known as binding immunoglobin protein
CHOP: CCAAT/enhancer binding homologous protein
EDEM: ER-associated degradation
*EPO*: Erythropoietin
ER: Endoplasmic reticulum
FIH: Factor inhibiting HIF
GADD34: Growth arrest and DNA damage inducible protein
GRP78: Glucose-regulated protein 78
HIFs: Hypoxia-inducible factors
HREs: Hypoxia-response elements
IRE1α: The inositol-requiring enzyme 1α
PERK: Protein kinase RNA-like endoplasmic reticulum kinase
PHDs: Proline-hydroxylases
RIDD: IRE1-dependent decay
ROS: Reactive oxygen species
UPR: Unfolded protein response
VEGF: Vascular endothelial growth factor
